# Compromised SARS-CoV-2-specific placental antibody transfer

**DOI:** 10.1016/j.cell.2020.12.027

**Published:** 2021-02-04

**Authors:** Caroline Atyeo, Krista M. Pullen, Evan A. Bordt, Stephanie Fischinger, John Burke, Ashlin Michell, Matthew D. Slein, Carolin Loos, Lydia L. Shook, Adeline A. Boatin, Laura J. Yockey, David Pepin, Marie-Charlotte Meinsohn, Ngoc Minh Phuong Nguyen, Maeva Chauvin, Drucilla Roberts, Ilona T. Goldfarb, Juan D. Matute, Kaitlyn E. James, Lael M. Yonker, Lisa M. Bebell, Anjali J. Kaimal, Kathryn J. Gray, Douglas Lauffenburger, Andrea G. Edlow, Galit Alter

**Affiliations:** 1 Ragon Institute of MGH, MIT, and Harvard, Cambridge, MA 02139, USA; 2 PhD Program in Virology, Division of Medical Sciences, Harvard University, Boston, MA 02115, USA; 3 Department of Biological Engineering, Massachusetts Institute of Technology, Cambridge, MA 02139, USA; 4 Department of Pediatrics, Lurie Center for Autism, Massachusetts General Hospital, Harvard Medical School, Boston, MA 02114, USA; 5 PhD Program in Immunology and Virology, University of Duisburg–Essen, Essen 47057, Germany; 6 Department of Obstetrics, Gynecology and Reproductive Biology, Massachusetts General Hospital, Harvard Medical School, Boston, MA 02114, USA; 7 Department of Medicine, Massachusetts General Hospital, Harvard Medical School, Boston, MA 02114, USA; 8 Pediatric Surgical Research Laboratories, Department of Surgery, Massachusetts General Hospital, Harvard Medical School, Boston, MA 02114, USA; 9 Department of Pathology, Massachusetts General Hospital, Harvard Medical School, Boston, MA 02114, USA; 10 Department of Pediatrics, Massachusetts General Hospital, Harvard Medical School, Boston, MA 02114, USA; 11 Division of Infectious Diseases, Massachusetts General Hospital, MGH Global Health, and Harvard Medical School, Boston, MA 02114, USA; 12 Department of Obstetrics, Gynecology and Reproductive Biology, Brigham and Women’s Hospital, Harvard Medical School, Boston, MA 02115, USA; 13 Vincent Center for Reproductive Biology, Massachusetts General Hospital, Boston, MA 02114, USA

**Keywords:** SARS-CoV-2, pregnancy, placental transfer, Fc-receptor, glycosylation, antibodies, fucose, hypergammablobulinemia, infection, trimester, inflammation

## Abstract

SARS-CoV-2 infection causes more severe disease in pregnant women compared to age-matched non-pregnant women. Whether maternal infection causes changes in the transfer of immunity to infants remains unclear. Maternal infections have previously been associated with compromised placental antibody transfer, but the mechanism underlying this compromised transfer is not established. Here, we used systems serology to characterize the Fc profile of influenza-, pertussis-, and SARS-CoV-2-specific antibodies transferred across the placenta. Influenza- and pertussis-specific antibodies were actively transferred. However, SARS-CoV-2-specific antibody transfer was significantly reduced compared to influenza- and pertussis-specific antibodies, and cord titers and functional activity were lower than in maternal plasma. This effect was only observed in third-trimester infection. SARS-CoV-2-specific transfer was linked to altered SARS-CoV-2-antibody glycosylation profiles and was partially rescued by infection-induced increases in IgG and increased FCGR3A placental expression. These results point to unexpected compensatory mechanisms to boost immunity in neonates, providing insights for maternal vaccine design.

## Highlights

• SARS-CoV-2-specific antibodies have a decreased placental transfer
• SARS-CoV-2-spike antibodies have altered glycosylation profiles in pregnant women
• Pregnant women with SARS-CoV-2 during the third trimester have elevated IgG levels
• SARS-CoV-2-specific antibody transfer is efficient after second-trimester infection

## Graphical abstract

**Figure:**
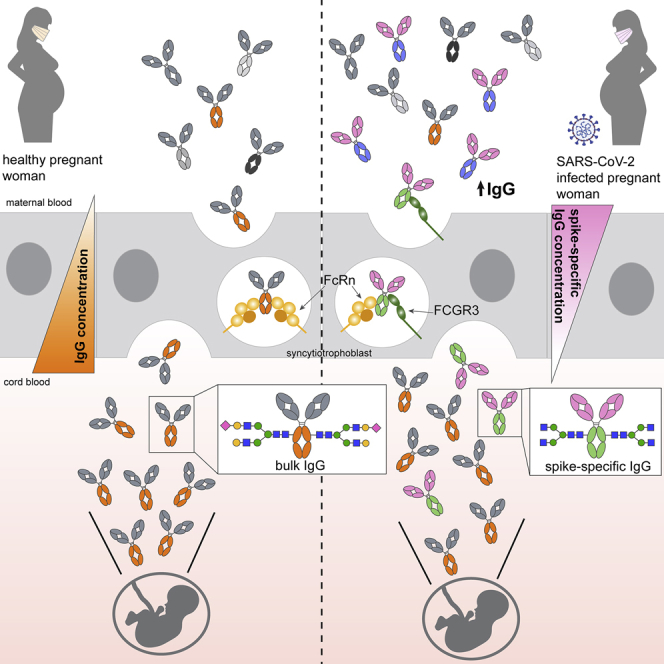

## Teaser

Atyeo et al. reveal a deficiency in SARS-CoV-2-antibody placental transfer among women infected during the third trimester. While bulk antibody transfer remains unaltered, SARS-CoV-2-antibodies show perturbed Fc glycosylation profiles and elevated IgG and FCGR3A placental expression that suggest compensation for poor transfer.

## Introduction

Over 44,000 pregnant women in the U.S. have been infected with SARS-CoV-2, and, with an estimated 140 million births annually worldwide, the number of pregnant women infected this year alone is likely in the millions (CDC, 2020). Although up to 16% of pregnant women test positive for SARS-CoV-2 in geographic hotspots (Breslin et al., 2020; Sutton et al., 2020), pregnant women and neonates are excluded from vaccine and therapeutic trials due to enhanced safety standards required for this population. Previous work has shown that both newborns and pregnant women are particularly susceptible to respiratory infections, including influenza and respiratory syncytial virus (RSV) (Zaman et al., 2008; Rasmussen et al., 2012; Gerretsen and Sande, 2017; Reeves et al., 2019; Liu et al., 2020). Recent data demonstrate that a greater proportion of neonates and infants have severe or critical illness upon SARS-CoV-2 infection compared to older pediatric counterparts (Kim et al., 2020; Dong et al., 2020). Given the immature nature of the newborn’s immune system, coupled with anticipated delays in vaccine deployment to pregnant women and children, infants are highly vulnerable during the SARS-CoV-2 pandemic.

Neonates rely on the transfer of maternal immunoglobulin G (IgG) across the placenta for protection against pathogens. For most pathogens, umbilical cord titers of IgG are higher than in maternal blood (Gonçalves et al., 1999; Munoz et al., 2014; Martinez et al., 2019), due to endosomal transport of IgG across the syncytiotrophoblast cell barrier from maternal to fetal circulation (Firan et al., 2001). These antibodies are transferred by the neonatal Fc receptor (FcRn), which is found in high concentrations on the placental syncytiotrophoblast (Leach et al., 1996; Simister et al., 1996). Placental IgG transfer begins during the first trimester but increases exponentially during pregnancy, with the majority of transfer occurring during the third trimester (Fouda et al., 2018). Recent studies point to selective transfer of IgG across the maternal-fetal interface based on subclass (Palmeira et al., 2012; Wilcox et al., 2017; Langel et al., 2020) and Fc-glycan profile (Jennewein et al., 2019; Martinez et al., 2019; Langel et al., 2020). Across the IgG subclasses, IgG1 antibodies are transferred preferentially, followed by IgG3, IgG2, and IgG4 (Palmeira et al., 2012; Vidarsson et al., 2014). Antibody glycosylation, a post-translational modification, impacts the transfer of IgG across the placenta. Among IgG1 antibodies, galactosylated antibodies are transferred preferentially, potentially as a result of enhanced binding to both placental FcRn and FCGR3 (Kibe et al., 1996; Jennewein et al., 2019), enabling the selective transfer of specific antibody subpopulations to arm neonates most effectively in the setting of pathogen exposure. Recent reports have demonstrated infection-induced changes of Fc-glycan profiles in SARS-CoV-2-infected individuals (Chakraborty et al., 2020), raising the possibility that SARS-CoV-2 infection during pregnancy influences the quality of transferred immunity. However, the impact of altered glycosylation on maternal-to-neonatal antibody transfer remains unclear.

Maternal infection may alter the ability of antibodies to transfer across the placenta, in part by altering glycosylation. Prior studies have found that both maternal HIV and malaria infection result in reduced placental transfer of non-disease-specific antibodies (Okoko et al., 2001; Farquhar et al., 2005; Cumberland et al., 2007; Ogolla et al., 2015). This compromised transfer has been attributed to infection-associated alterations in antibody glycosylation (Martinez et al., 2019) and to hypergammaglobulinemia in the infected mother, resulting in competition for binding to FcRn on the placenta (Englund, 2007). Less is known about placental transfer of disease-specific antibodies in the setting of maternal acute infection. Maternal:cord transfer ratios of ~1.0 have been noted in maternal Dengue (DENV) or Zika (ZIKV) acute viral infections (Perret et al., 2005; Castanha et al., 2019) in contrast to ratios of 1.5 or greater typically observed for vaccinatable pathogens, such as influenza and pertussis (Gonçalves et al., 1999; Heininger et al., 2009; Munoz et al., 2014; Castanha et al., 2019; Singh et al., 2019; Collier et al., 2020). These lower-than-expected transfer ratios suggest that features of *de novo* antibodies generated in the setting of acute infection, such as glycosylation profile, may drive less efficient placental transfer. However, the precise mechanism of these alterations in placental antibody transfer in the setting of recent infection is not known and has yet to be elucidated in maternal SARS-CoV-2 infection.

To address these gaps, we profiled the influenza, pertussis, and SARS-CoV-2 humoral immune response across 22 matched maternal:cord dyads from mothers who tested positive for SARS-CoV-2 infection in the third trimester of pregnancy (COVID^+^) and 34 contemporaneously enrolled maternal-cord dyads that were SARS-CoV-2 negative (COVID^–^) using systems serology (Chung et al., 2015). Influenza hemagglutinin (HA)- and pertussis pertactin (PTN)-targeting antibodies were transferred efficiently from COVID^–^ mothers and COVID^+^ mothers. In contrast, we observed significantly decreased transfer of SARS-CoV-2-specific IgG and antibody functions across multiple SARS-CoV-2 specificities compared to HA. While similar glycan-dependent bulk (total IgG) placental transfer was observed across COVID^–^ and COVID^+^ dyads, compromised glycan-dependent transfer was noted for SARS-CoV-2-specific antibodies. In addition, while higher IgG levels in COVID^–^ women were associated with compromised placental transfer of HA-specific antibodies, increases in total IgG and placental co-localization of FCGR3A with FcRn played compensatory roles in augmenting SARS-CoV-2 antibody transfer across the COVID^+^ dyads. These data provide mechanistic insights into the changes in antibody glycosylation and placental Fc-receptor expression that contribute to compromised third-trimester antibody transfer to infants, pointing to opportunities to bolster neonatal immunity to SARS-CoV-2 and beyond.

## Results

### SARS-CoV-2-specific antibodies are transferred inefficiently to the neonate

**Figure 1:**
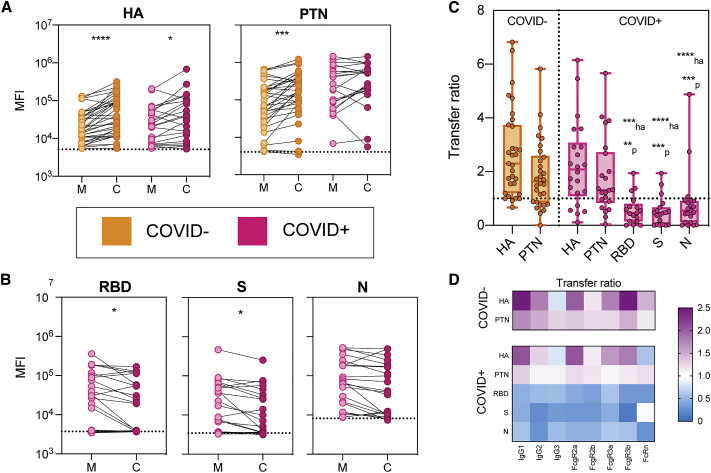
Inefficient placental transfer of SARS-CoV-2 antibodies (A) The dot plots show the relative hemagglutinin- (HA) (left) and pertactin- (PTN) (right) specific IgG1 titers for both COVID-negative (COVID^–^, orange) and COVID-positive (COVID^+^, pink) maternal-cord pairs. The dotted line indicates background PBS levels. Significance was determined by a Wilcoxon signed-rank test, ^∗^p < 0.05, ^∗∗∗^p < 0.001, ^∗∗∗∗^p < 0.0001. (B) The dot plot shows the IgG1 titer against SARS-CoV-2 receptor binding domain (RBD, left), spike (S) (middle), and nucleocapsid (N) (right). The lines connect maternal:cord pairs. The dotted line shows the PBS background level. Significance was determined by Wilcoxon signed-rank test, ^∗^p < 0.05. (C) The dot plots show the transfer ratio of HA, PTN, RBD, S, and N antibodies. The transfer ratio of COVID^–^ dyads are shown in orange, whereas the transfer ratios of COVID^+^ dyads are shown in pink. The horizontal dotted line indicates a 100% transfer efficiency (equivalent levels across both compartments). Significance was determined by using a one-way ANOVA followed by Tukey’s multiple comparisons test, where “ha” indicates a significant difference compared to the COVID^+^ HA transfer ratio and “p” indicates a significant difference compared to the COVID^+^ PTN transfer ratio. ^∗∗^p < 0.01, ^∗∗∗^p < 0.001, ^∗∗∗∗^p < 0.0001. (D) The heatmap shows the median PBS-background corrected transfer ratio of HA, PTN, RBD, S, and N across all antibody subclasses and FcR binding profiles. See also Figures S1, S2, and S3.

**Figure S1:**
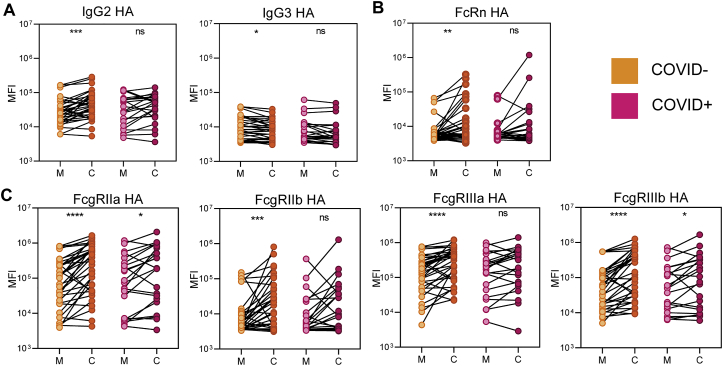
HA-specific antibodies are increased in the cord across subclasses and FcR-binding profiles, related to Figure 1 **A-C.** The dot plots show the relative HA-specific IgG2 (A, right), IgG3 (A, left), FcRn-binding (B), and FcgR-binding (C) in maternal plasma (M) and cord blood (C) for COVID- dyads (left, orange) and COVID+ dyads (right, pink). Lines connect maternal (M):cord (C) pairs. Significance was determined by a Wilcoxon signed rank test: ns is not significant, ^∗^p < 0.05, ^∗∗^p < 0.01, ^∗∗∗^p < 0.001, ^∗∗∗∗^p < 0.0001.

Past reports have described infection-driven alterations in placental IgG transfer to the neonate (Okoko et al., 2001; Farquhar et al., 2005; Cumberland et al., 2007; Ogolla et al., 2015; Martinez et al., 2019), but it is unclear whether placental transfer dynamics shift with SARS-CoV-2 infection. To address this question, we profiled humoral immune responses to three respiratory pathogens in COVID^+^ and COVID^–^ pregnant women and their neonates’ cord blood to gain a deeper understanding of neonatal immunity following maternal SARS-CoV-2 infection. Cohort demographics are depicted in Table 1. Mean time from symptom onset to delivery was 30.4 ± 19.8 days. No neonates were infected with SARS-CoV-2; thus, COVID^+^ infection status throughout the manuscript refers only to the mother (Edlow et al., 2020). We first compared the relative titer of antibodies in maternal plasma and cord blood against influenza hemagglutinin (HA) and pertussis pertactin (PTN). As expected, increased titers of HA- and PTN-specific IgG1 were observed in the cord compared to maternal blood in both COVID^–^ and COVID^+^ mother:cord dyads (Figure 1A). Similarly, all HA-specific subclasses and Fc receptor (FcR) binding were increased in cord blood compared to the maternal plasma across the dyads, with the exception of IgG3, which has been shown to have low affinity for FcRn compared to other IgG subclasses (Vidarsson et al., 2014) (Figure S1). These data demonstrate conserved efficient transfer of HA- and PTN-specific antibodies in the setting of SARS-CoV-2 infection, albeit less significant skewing (lower p value differences) of antibodies in the cord in COVID^+^ dyads across the placenta (Figures 1A and S1).

**Table 1:** Demographics of cases and controls

	COVID Negative (n = 33)^a^	COVID Positive (n = 22)^b^	P Value^c^
Maternal age (median, IQR)	33 (30, 37)	33 (28, 39)	0.91
Gestational age (mean, SD)	39.2 (1.5)	38.0 (4.1)	0.16
Parity (median, IQR)	1 (0, 2)	1 (0, 1)	0.65
Race			0.02
Asian	2 (6%)	0 (0%)	
Black/African American	0 (0%)	2 (9%)	
White	26 (79%)	10 (45%)	
Other	4 (12%)	4 (18%)	
More than one race	0 (0%)	2 (9%)	
Unknown/not reported	1 (3%)	4 (18%)	
Ethnicity			0.02
Hispanic or Latino	7 (21%)	13 (59%)	
Not Hispanic or Latino	24 (73%)	8 (36%)	
Unknown/Not Reported	2 (6%)	1 (5%)	
Severity^d^			
Asymptomatic	–	6 (27%)	
Mild	–	9 (41%)	
Moderate	–	3 (14%)	
Severe	–	4 (18%)	
Time from symptom onset to delivery, in days (median, IQR)	–	30.5 (8.5, 44)	N/A
Time from last influenza vaccination, in days (median, IQR)	200 (178, 209)	204 (188.5, 215.5)	0.82
Time from last pertussis vaccination, in days (median, IQR)	69 (51.75, 81.25)	72 (57, 81)	0.31

a n = 33 COVID negative (34 dyads, one mother of di-di twins).

b No neonates tested positive for SARS-CoV-2 by RT-PCR of nasopharyngeal swab.

c Significant differences between groups were determined using Pearson’s chi-square test for categorical variables, and Mann-Whitney U test or Student’s t test for continuous variables (median, IQR and mean, SD, respectively).

d Severity determinations were made based on published criteria from the Society for Maternal-Fetal Medicine and the National Institutes of Health.

**Figure S2:**
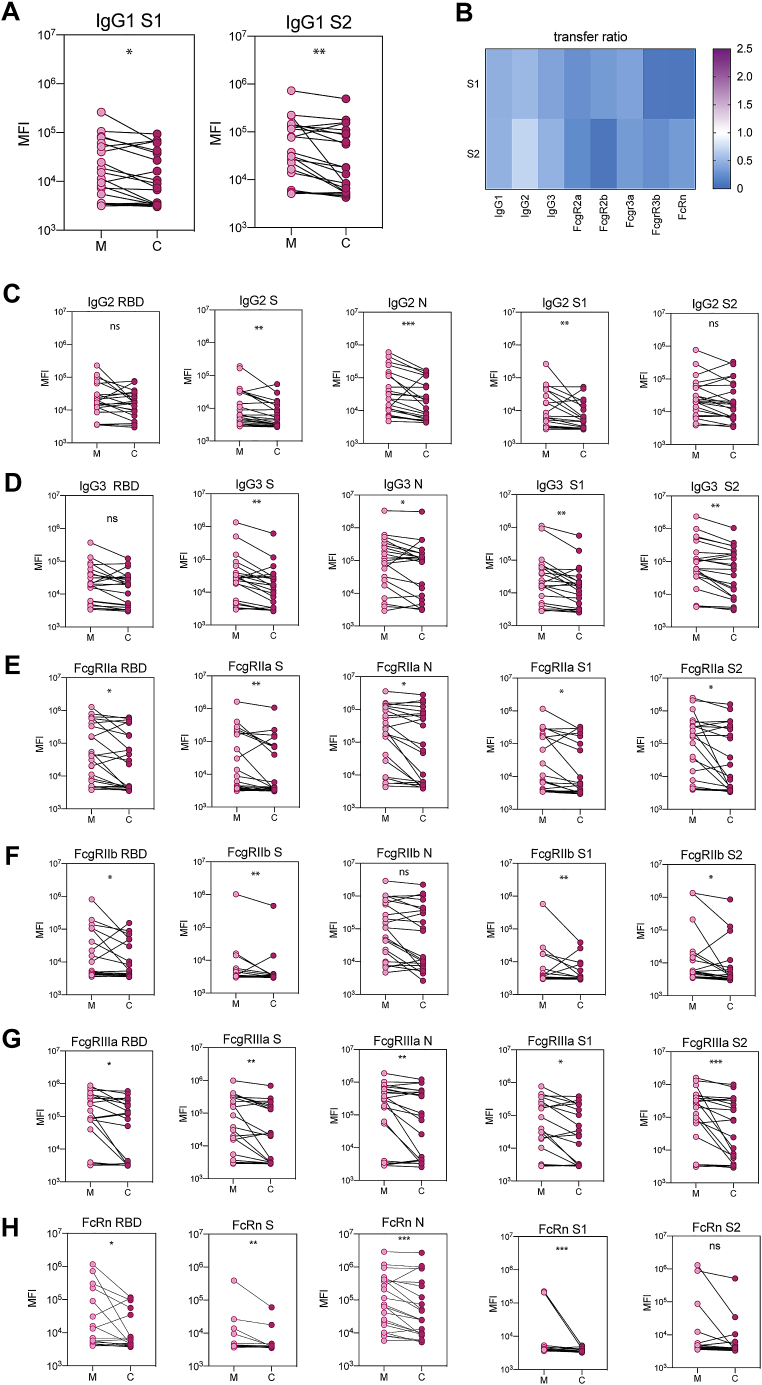
SARS-CoV-2-targeting antibodies are transferred inefficiently to the cord across subclasses and FcR binding, related to Figure 1 **A.** The dot plots show the titer of IgG1 against SARS-CoV-1 S1 and S2 in SARS-CoV-2 infected dyads. Lines connect maternal (M):cord (C) pairs. Significance was determined by a Wilcoxon signed rank test, ^∗^p < 0.05, ^∗∗^p < 0.01, ^∗∗∗^p < 0.001, ^∗∗∗∗^p < 0.0001. **B.** The heatmap shows the median transfer ratio of SARS-CoV-2 S1 and S2-targeting IgG and FcR-binding. **C-H.** The dot plots show the titer of IgG2 (C), IgG3 (D), and FcR binding (E-H) against RBD, spike (S), nucleocapsid (N), S1 and S2 in maternal plasma (M) and cord blood. Lines connect maternal (M):cord (C) pairs. Significance was determined by a Wilcoxon signed rank test, ^∗^p < 0.05, ^∗∗^p < 0.01, ^∗∗∗^p < 0.001, ^∗∗∗∗^p < 0.0001.

**Figure S3:**
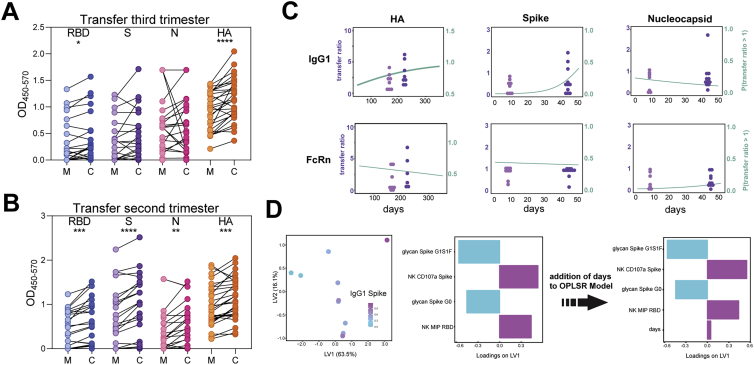
The effect of time on SARS-CoV-2-specific antibody transfer, related to Figures 1 and 4 **A-B**. The dot plots show the relative IgG titer for RBD, S, N and HA in maternal plasma and cord blood in which the mother was infected during the third (A) or second (B) trimester. Lines connect mother:cord dyads. The data is represented as the absorbance at 450 nm subtracted by the reference absorbance read at 570 nm. The data was background corrected using plasma that was negative for SARS-CoV-2 antibodies. (**C**). Transfer ratios for IgG1 and FcRn were plotted according to time from last vaccination for HA (left) or from symptom onset or positive test for SARS-CoV-2 S and N (right), using a median split based on time. Logistic regression was performed to calculate the probability of each transfer ratio to be greater than 1 based on time (green line). **D.** An orthogonal partial least square regression (OPLSR) was built using SARS-CoV-2-specific antibody features and spike-specific glycan data with IgG1 spike transfer ratio as the outcome variable. The scores plot is colored based on IgG1 spike transfer ratio (left). When time (or days since symptom onset/positive test result) was added to the Elastic Net reduced feature set, the loadings of the previously selected features changed minimally and the Elastic Net selected features, rather than time elapsed since infection, continued to drive IgG1 spike transfer ratio prediction (right).

To determine whether the same pattern was present for SARS-CoV-2-specific antibodies, we profiled levels of SARS-CoV-2 receptor binding domain (RBD), spike (S), and nucleocapsid (N)-specific responses across the COVID^+^ mother:cord pairs (Figures 1B and S2). Surprisingly, rather than the expected active transfer to the cord, resulting in higher cord titers compared to maternal titers, reduced titers of RBD- and S-specific antibodies were present in the cord, and N-specific titers were stable or slightly reduced in the cord. To further compare changes in transfer across all antigen specificities and dyads, we plotted IgG1 transfer ratios against each antigen by maternal infection status (Figure 1C). Expected transfer ratios (>1) were observed for HA- and PTN-specific antibodies in COVID^–^ mothers. In contrast, a significant defect in transfer was visualized across antibody subclasses and FcR-binding to RBD, S, and N, including the S1 and S2 domain of the S protein (Figure 1C, 1D, and S2). In addition, while enhanced transfer of antibodies able to bind FcRs was observed for HA and PTN, there was a reduced transfer of SARS-CoV-2-specific, FcR-binding antibodies (Figures S1 and S2). Moreover, in a second independent set of mother:cord pairs, compromised SARS-CoV-2 antibody transfer was confirmed in mothers infected during the third trimester (Figure S3A). Interestingly, this defect was exclusive to third-trimester infection, as efficient SARS-CoV-2 antibody transfer was observed for mothers infected during the second trimester (Figure S3B). In addition, in the third-trimester-infected cohort, time from infection had no significant effect on transfer rates (Figure S3C). Collectively, these data show that maternal third-trimester SARS-CoV-2 infection has a profound impact on SARS-CoV-2-specific neonatal antibody transfer compared to transfer of other pathogen-specific antibodies.

### Compromised SARS-CoV-2-specific functional antibody transfer

**Figure 2:**
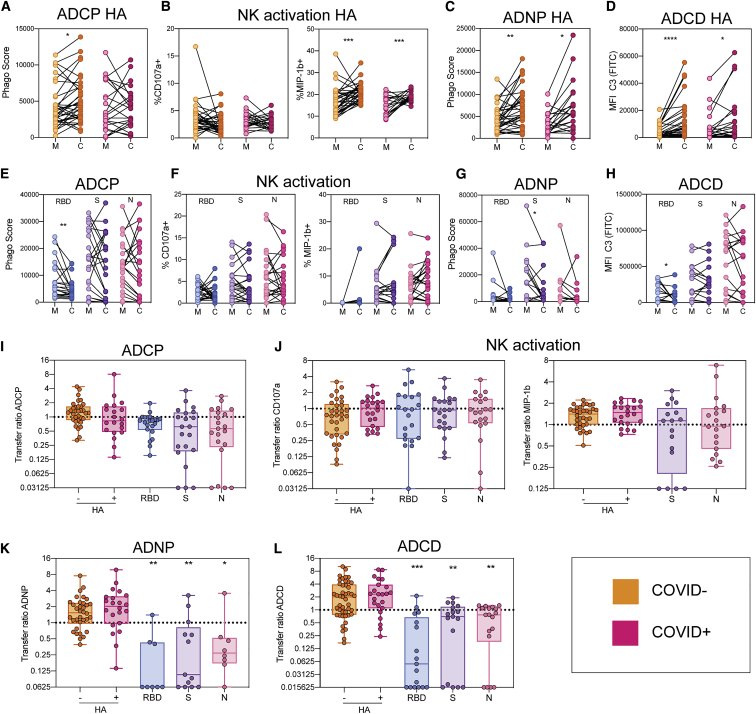
Functional HA-, but not SARS-CoV-2-, specific antibodies are transferred efficiently across the placenta (A–D) The dot plots illustrate antibody-dependent monocyte phagocytosis (ADCP, A), natural killer cell (NK) activation (B), antibody-dependent neutrophil phagocytosis (ADNP, C), and antibody-dependent complement deposition (ADCD, D) (PBS background corrected) in maternal plasma (M) and cord-blood plasma (C) against hemagglutinin (HA). Lines connect maternal:cord pairs. COVID^–^ dyads are represented in orange, and COVID^+^ dyads are represented in pink. Significance was determined by a Wilcoxon signed-rank test, ^∗^p < 0.05, ^∗∗^p < 0.01, ^∗∗∗^p < 0.001, ^∗∗∗∗^p < 0.0001. (E–H) The dot plots show the ADCP (E), NK activation (F), ADNP (G), and ADCD (H) activity (PBS background corrected) in maternal blood (M) and cord plasma (C) for the SARS-CoV-2 antigens RBD, spike (S), and nucleocapsid (N). Lines connect maternal:cord dyads. Significance was determined by a Wilcoxon signed-rank test, ^∗^p < 0.05, ^∗∗^p < 0.01. (I–L) The box-and whisker plots show the transfer-ratios for ADCP (I), NK activation (J), ADNP (K), and ADCD (L) against HA, RBD, S, and N. For HA, COVID^–^ dyads are represented in orange and COVID^+^ dyads are shown in pink. For each SARS-CoV-2 antigen, significance was determined against the HA activity using the matched COVID^+^ dyad. A one-way ANOVA followed by Tukey’s multiple comparison test was performed to determine significance, ^∗^p < 0.05, ^∗∗^p < 0.01, ^∗∗∗^p < 0.001.

To further dissect the impact of SARS-CoV-2 infection on antibody transfer, we examined whether preferential transfer occurred for any humoral immune function across the dyads. Similar to titer, increased HA-specific functional antibodies were transferred to the cord across both the COVID^–^ and COVID^+^ pairs (Figures 2A–2D). The only exceptions were the transfer of antibodies that induce monocyte phagocytosis (ADCP), which were not preferentially transferred in COVID^+^ mothers (Figure 2A), and natural killer (NK) cell-degranulation-inducing antibodies (CD107a) that were transferred less efficiently across COVID^+^ and COVID^–^ dyads (Figure 2B). HA-specific NK cell chemokine secretion- (macrophage inflammatory protein-1b or MIP-1b), neutrophil phagocytosis- (ADNP), and complement deposition (ADCD)-inducing antibodies were transferred efficiently across both COVID^+^ and COVID^–^ dyads (Figures 2B–2D). In contrast, significantly reduced or erratic transfer was noted for RBD, S, and N-specific antibody functions in COVID^+^ dyads (Figures 2E–2H). For SARS-CoV-2 specificities, there was a decrease, though not always significant, in functional antibodies able to drive ADCP, ADNP, and ADCD in cord blood compared to maternal plasma (Figures 2E, 2G, and 2H). In contrast, the levels of SARS-CoV-2-specific NK cell-activating antibodies were not significantly reduced between maternal plasma and cord blood (Figure 2F).

To determine whether antibodies with certain functions were selectively transferred, a transfer ratio was calculated for each specificity and function, where a transfer ratio of 1 denoted equivalency in antibody effector function across the maternal:cord pairs. Collectively, median transfer ratios for HA-directed functions were above 1. No significant differences were noted in the transfer ratios for HA-directed functions between COVID^+^ and COVID^–^ dyads, indicating more efficient transfer of HA-directed functions compared to SARS-CoV-2-directed functions (Figures 2I–2L). The median transfer ratios for all functions except NK activation were below 1 for SARS-CoV-2 specificities, indicating a decrease, although not always significant, in the transfer of antibodies able to drive ADNP, ADCD, and ADCP against SARS-CoV-2 in the cord. The higher transfer ratio of SARS-CoV-2 NK cell chemokine secretion-inducing antibodies (MIP-1b) (Figure 2J), despite lower SARS-CoV-2-specific IgG levels in the cord (Figure 1), suggests conserved preferential placental transfer of these antibodies to the neonates compared to antibodies able to drive neutrophil and complement activation (Figures 2K and 2L, respectively), as has been shown previously (Jennewein et al., 2019). Taken together, these findings point to relatively normal placental antibody transfer of non-SARS-CoV-2 antibodies in the setting of maternal SARS-CoV-2 infection but a distinct loss of neutrophil phagocytosis- and complement-inducing SARS-CoV-2-specific functional antibody transfer, two functions that have been linked to enhanced antiviral control following natural infection in adults (Atyeo et al., 2020).

### Altered SARS-CoV-2-specific glycan profiles shift placental transfer profiles

**Figure 3:**
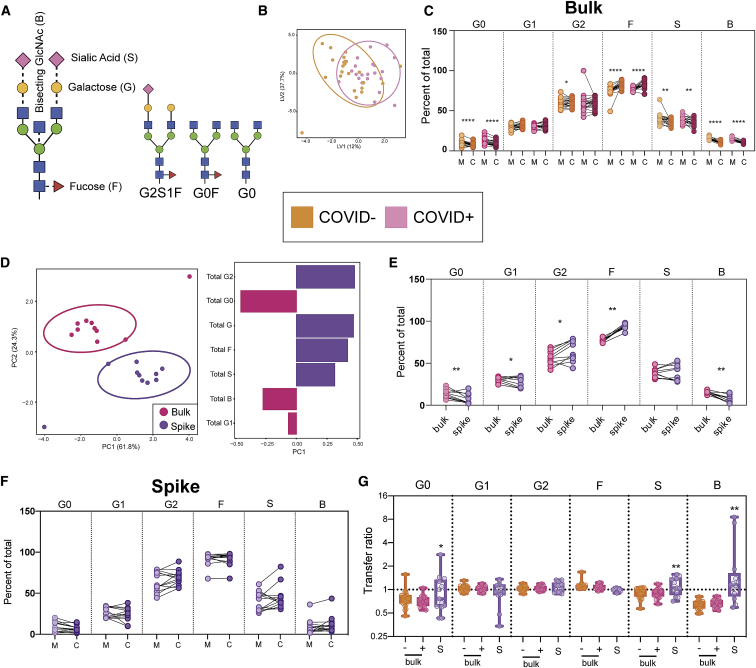
Spike-specific Fc-glycan profiles diverge from bulk antibody profiles (A) The figure represents a typical Fc glycan. The solid lines between sugars represent the linkages that are always present, whereas the dotted lines represent sugars that can be added. (B) An orthogonal partial least-squares discriminant analysis (OPLSDA) model was built using bulk glycan features from COVID^–^ (orange) and COVID^+^ (pink) maternal plasma samples. This orthogonal approach ensures that maximal variance between groups is captured in the first latent variable (LV1). The dot plot shows the distribution of each maternal sample, with ellipses encompassing the 95% confidence interval for each group. (C) The dot plot shows the percent of each glycoform in bulk antibodies in maternal (M) and cord (C) samples. The glycoforms are denoted as follows: agalactosylated (G0), monogalactosylated (G1), digalactosylated (G2), fucosylated (F), sialylated (S), and bisected n-acetyl-glucosamine (GlcNAc) (B). Connected lines signify maternal:cord dyads. Significance was determined using a Wilcoxon signed-rank test, ^∗^p < 0.05, ^∗∗^p < 0.01, ^∗∗∗^p < 0.001, ^∗∗∗∗^p < 0.0001. (D) A multi-level principal component analysis (m-PCA) was built using bulk (pink) and spike-specific (purple) antibody glycan data from COVID^+^ mothers for which matched data were available. Each maternal sample is represented as a dot on the scores plot (left). The bar graph (right) shows the loadings on principal component 1 (PC1) of each glycan feature. (E) The dot plot shows the percentage of each glycoform in maternal bulk (pink) and spike-specific (purple) antibodies. The lines represent glycan data from the same mother. Significance was determined using a Wilcoxon signed-rank test, ^∗^p < 0.05, ^∗∗^p < 0.01, ^∗∗∗^p < 0.001, ^∗∗∗∗^p < 0.0001. (F) The dot plot shows the percent of each glycoform in spike-specific antibodies in maternal (M) and cord (C) samples. Significance was determined using a Wilcoxon signed-rank test (G) The boxplots show the transfer ratio of each glycoform for bulk (orange/pink) and spike-specific (purple) antibodies. For bulk glycan data, COVID^–^ dyads are represented in orange and COVID^+^ dyads are represented in pink. Spike (S)-specific glycan data are shown in purple. Significance was determined by a Mann-Whitney test between COVID^+^ bulk and S-specific antibodies for each glycan feature, ^∗^p < 0.05, ^∗∗^p < 0.01, ^∗∗∗^p < 0.001, ^∗∗∗∗^p < 0.0001. See also Figure S4.

**Figure S4:**
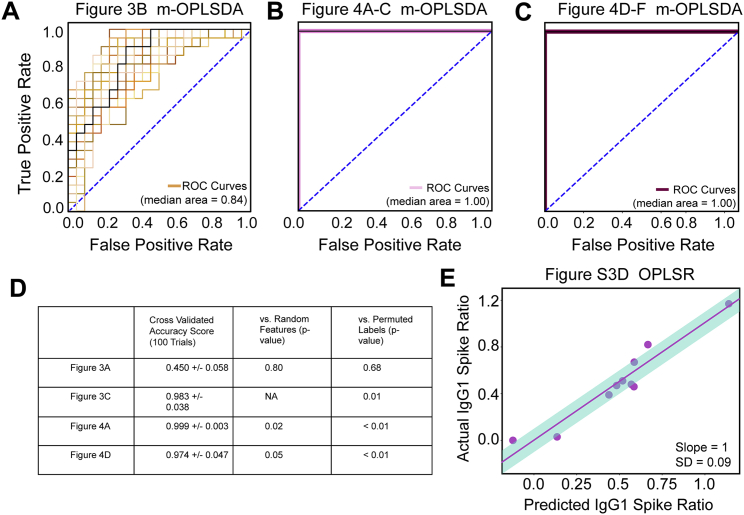
Multivariate model validation, related to Figures 3 and 4 **A-C**. ROC curves corresponding to m-OPLSDA models in (**A**) Figure 3B, (**B**) Figures 4A–4C, and (**C**) Figures 4D–4F. 100 trials of 5-fold CV were run for each model, illustrated by colored curves. The ROC curve corresponding with the median AUC score was plotted in black with the numerical score reported in the legend. The blue dashed line indicates the classification threshold for a random process. D. Table of performance metrics for each OPLSDA model, including mean accuracy score (left) and comparison models generated with random features (center) or permuted disease state labels (right). In each case, 100 trials were performed with 5-fold cross validation. (**E**). Plot of actual-versus-predicted transfer ratios for the OPLSR models Figure S3D. The solid line represents the linear regression of the data and the shaded area represents the standard deviation (SD) of the slope of the regression line when leave-one-out cross validation was performed. Slope approaching the value of 1 indicates good model performance.

Given the emerging role of antibody Fc glycosylation in placental transfer efficiency (Jennewein et al., 2019; Martinez et al., 2019), we next probed whether changes in SARS-CoV-2 antibodies themselves could account for differences in transfer efficiencies. Fc glycosylation (Figure 3A) was captured on bulk and SARS-CoV-2-S-specific antibodies. Bulk antibody Fc-glycan profiles were indistinguishable across COVID^+^ and COVID^–^ mothers (Figures 3B, S4A, and S4D), with similar bulk Fc glycosylation transfer patterns across COVID^+^ and COVID^–^ dyads (Figure 3C), consistent with previously observed selection of galactosylated Fc glycans across the placenta (Jennewein et al., 2019; Borghi et al., 2020). The conserved bulk antibody transfer profiles across the COVID^+^ and COVID^–^ dyads point to the preserved ability of the placenta to preferentially transfer IgG with certain glycosylation features in the setting of SARS-CoV-2 infection.

Antigen-specific antibody subpopulations may harbor distinct antibody Fc-glycan profiles compared to bulk circulating antibodies (Mahan et al., 2016). Thus, to determine whether there were SARS-CoV-2-antibody-specific changes to Fc-glycan profiles, we next compared spike-specific Fc glycosylation to bulk antibodies across the COVID^+^ mothers. In contrast to the bulk antibody Fc profiles (Figure 3B), which did not differ across mothers, spike (S)-specific Fc-glycan profiles were significantly different within COVID^+^ mothers compared to bulk Fc-glycan profiles (Figure 3D, left), marked by enhanced digalactosylation (G2), fucosylation (F), and reduced agalactosylation (G0) and bisecting-n-acetyl-glucosamine (b-GlcNAc, B) on S-specific antibodies (Figures 3D, right, and 3E). As expected, these glycan-profile differences resulted in altered antibody transfer (Figure 3F). Consistent with bulk transfer glycan profiles, digalactosylated (G2) antibody transfer efficiency was above 1 for S-specific transfer (Figures 3F and 3G). Transfer ratios were perturbed for agalactosylated (G0), sialyated (S), and b-GlcNAc (B) levels on S-specific antibodies compared to bulk antibody transfer in the COVID^+^ and COVID^–^ dyads (Figure 3G). Increased transfer of G0 S-specific antibodies relative to bulk antibodies in COVID^+^ dyads may suggest preferential transfer of inflammatory afucosylated, bisected, G0 antibodies in the setting of maternal SARS-CoV-2 infection (Figure 3G). However, whether perturbed glycan transfer profiles were related to altered placental transfer of SARS-CoV-2 antibodies, or simply linked to the overall availability of different Fc-glycan profiles on maternal SARS-CoV-2-specific antibodies, remained unclear.

### Signatures of FCGR3A-selective transfer in COVID^+^ dyads

**Figure 4:**
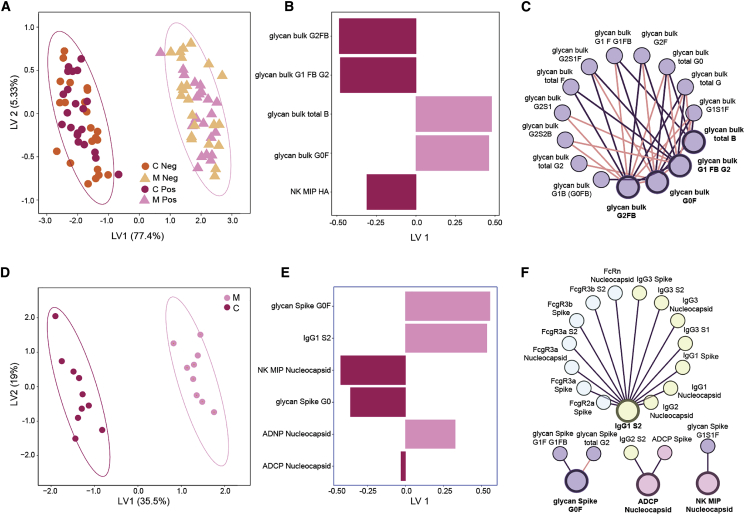
Placental sieving signatures of HA- and spike-specific antibodies (A) Multi-level orthogonal partial least square discriminant analysis (m-OPLSDA) was performed to distinguish between profiles from mother and cord plasma using HA, PTN, and bulk glycan data. Triangular and circular data points on the scores plot represent mother and cord samples, respectively. Infection status is indicated by color (orange representing COVID^–^ and pink representing COVID^+^ individuals). The ellipses show the 95% confidence interval for the mother/cord populations. (B) The bar graph illustrates the loadings on latent variable 1 (LV1) of the Elastic Net-selected antibody features from the m-OPLSDA shown in (A). The color indicates the group in which each feature is enriched (dark pink for cord samples, light pink for maternal plasma). (C) A pairwise spearman correlation test was performed to determine features that correlate with the Elastic Net-selected features (bolded nodes) selected for the m-OPLSDA model shown in (A). Features with correlation of coefficients >|0.75| and p < 0.05 are shown in the networks. Positive correlations are shown in purple, and negative correlations are shown in pink. Nodes are colored by features type, with glycan data shown in purple, antibody titer shown in yellow, and FcR binding shown in blue. (D) A m-OPLSDA model was built to classify COVID^+^ mother and cord plasma using SARS-CoV-2-specific antibody features and spike-specific glycan data. Only samples for which spike-specific glycan data were available were included in the model. The scores plot shows the separation between the cord samples (dark pink) and the maternal samples (light pink), where each dot represents a sample. The ellipses show the 95% confidence interval for each group. (E) The bar graph shows the loadings on latent variable 1 (LV1) of the Elastic Net-selected features for the m-OPLSDA model built in (D). The color indicates the group in which each feature is enriched (dark pink for cord samples, light pink for maternal samples). (F) A pairwise spearman correlation test was performed to determine features that were correlated with the Elastic Net-selected features (bold nodes) that were selected for the m-OPLSDA model shown in (D). Features with correlation of coefficients >|0.75| and p < 0.05 are shown in the networks. Positive correlations are shown in purple and negative correlations are shown in pink. Nodes are colored by feature type, with glycan data shown in purple, antibody titer shown in yellow, and FcR binding shown in blue. See also Figures S3 and S4.

To determine whether the placental transfer differences were simply related to different levels of glycosylated antibodies on the maternal side, or whether the transfer signatures were conserved across COVID^+^ and COVID^–^ mothers, we next examined the overall humoral Fc profiles of HA- and PTN-specific or non-specific antibodies in the setting of maternal SARS-CoV-2 infection or non-infection. To define distinct features between maternal plasma and cord blood across antibody populations, multivariate dimensionality reduction was coupled to multivariate visualization, using Elastic Net regularization and feature reduction with multi-level orthogonal partial least-squares discriminant analysis (m-OPLSDA). As expected, non-SARS-CoV-2-specific antibody profiles were distinct between maternal and cord blood (Figures 4A, S4B, and S4D), with a clear enrichment of digalactosyated antibodies and NK cell enhancing antibodies in the cord (Figure 4B). COVID^+^ and COVID^–^ samples were interspersed and not resolvable (Figure 4A), highlighting that SARS-CoV-2 infection does not alter the overall profile of transferred non-SARS-CoV-2-specific antibodies. Given that Elastic Net selects a minimal set of features in order to prevent overfitting of the data, we next generated a correlation network to determine additional features that correlated with the features included in the model (Figure 4C). Elastic Net-selected glycan features G2FB and G1FB-G2 were highly correlated with other galactosylated glycan features and negatively correlated with agalactosylated glycans. This bias toward highly galactosylated and fewer agalactosylated bulk antibodies in the cord emphasizes the importance of conserved glycan sieving in both COVID^+^ and COVID^–^ dyads.

Similar to HA, PTN, and bulk antibodies, SARS-CoV-2-specific antibody glycan profiles were clearly distinct in maternal versus cord blood (Figures 4D and S3C–3E). Maternal blood was enriched for fucosylated/agalactosylated S-specific antibodies (Spike G0F), S2-specific IgG1, and neutrophil functional antibodies, while the cord blood had elevated NK cell-activating nucleocapsid (N)-specific antibodies, S-specific afucosylated/agalactosylated (Spike G0), and N-specific monocyte phagocytic antibodies (Figure 4E). The enrichment of S2-domain-specific antibodies, recently found to cross-react across coronaviruses (Ng et al., 2020), was linked to a large S2- and S-specific functional network (Figure 4F). Given that IgG1 S2 was enriched in maternal blood, this network pointed to a lack of transfer of many SARS-CoV-2-specific antibodies, consistent with univariate data (Figures 1 and 2). The selective transfer of afucosylated/agalactosylated (G0) Fcs to the cord but the retention of fucosylated/agalactosylated (G0F) Fcs in the maternal circulation pointed to fucosylation-based antibody selection across the placenta, likely driven by FCGR3A, which preferentially binds to afucosylated glycans (Jennewein et al., 2019). Thus, consistent with previous data, the placenta appears to exhibit altered galactose transfer, but similar fucose transfer in COVID^+^ and COVID^–^ women, continuing to selectively sieve NK functional antibodies out of the SARS-CoV-2 maternal humoral response. Time from infection in third-trimester pregnancy contributed minimally to placental sieving (Figure S3E). These data suggest that reduced SARS-CoV-2-specific antibody transfer is not related to altered placental Fc-glycan sieving activity but rather to a bias against the glycoforms found dominantly on SARS-CoV-2-specific antibodies.

### Elevated IgG levels augment SARS-CoV-2 antibody transfer

**Figure 5:**
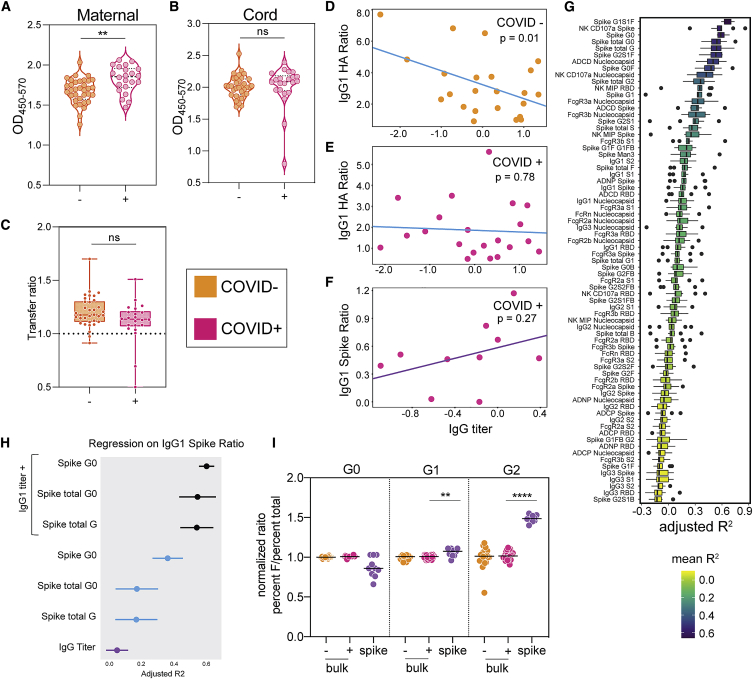
Elevated IgG compensates for glycosylation changes to improve SARS-CoV-2-specific antibody transfer (A and B) A total IgG ELISA was run on COVID^–^ (orange) and COVID^+^ (pink) maternal plasma (A) and cord blood (B). Significance was determined by a Mann-Whitney test. ^∗^p < 0.05, ^∗∗^p < 0.01, ^∗∗∗^p < 0.001, ^∗∗∗∗^p < 0.0001. (C) The boxplots show the transfer ratio, calculated as cord OD/maternal OD. The dotted line shows a transfer ratio of 1. Significance was determined by a Mann-Whitney test. (D–F) The dot plots illustrate the correlation between IgG titer and IgG1 HA (D and E) or IgG1 Spike (F) transfer ratio in COVID negative (orange) and COVID positive (pink) individuals. The direction of correlation is indicated by the color of the regression line (neg, blue; pos, purple). (G) 2-feature linear regression models were built to determine which feature, in combination with total IgG titer, was most predictive of SARS-CoV-2-specific antibody transfer ratio. The boxplots show the adjusted R^2^ values for the top performing models over 10 leave-one-out cross-validated trials. (H) The dots represent the adjusted R^2^ values of three of the top 2-feature models (G) to each of the individual features predictive performance of SARS-CoV-2-specific antibody transfer. For the 1-feature regression models, data points are colored by the direction of correlation with IgG1 spike transfer ratio (neg, blue; pos, purple). Error bars illustrate standard deviation of adjusted R^2^ after 10 rounds of leave-one-out cross-validation. (I) The dot plots represent the ratio of percent fucosylated to total percent for each galactose feature. For each galactose glycoform, the data were normalized by dividing by the average ratio of the bulk COVID^–^. Significance was only calculated between bulk COVID^+^ and spike-specific antibodies. Significance was determined by Mann-Whitney test, ^∗^p < 0.05, ^∗∗^p < 0.01, ^∗∗∗^p < 0.001, ^∗∗∗∗^p < 0.0001.

Despite evidence of conserved Fc-glycan selection across the placenta for SARS-CoV-2-specific antibodies, it remained unclear whether additional infection-related immune perturbations could influence transplacental transfer. Mounting evidence points to a critical role of infection-induced hypergammaglobulinemia in reducing antibody transfer (Okoko et al., 2001; Farquhar et al., 2005; Martinez et al., 2019), postulated to be due to increased competition for binding to FcRn and other Fc receptors important for placental transfer. Thus, we measured total IgG titer in maternal and cord blood to determine whether alterations in IgG levels tracked with changes in antibody transfer (Figures 5A and 5B). While the women in our study did not exhibit clinical hypergammaglobulinemia, we noted increased IgG levels in COVID^+^ mothers even weeks after infection. Total IgG titer was significantly higher in COVID^+^ mothers (Figure 5A) but not their cord blood (Figure 5B). Instead, total IgG transfer ratios trended lower in COVID^+^ mothers, highlighting the critical role of the placenta in creating a bottleneck to restrict antibody entry into the neonate (Figure 5C). Transfer efficiency for HA-specific antibodies was inversely correlated to IgG levels in COVID^–^ dyads (Figure 5D). Conversely, in COVID^+^ mothers, increasing IgG levels did not affect HA-IgG transfer (Figure 5E), suggesting a compensatory mechanism may exist during SARS-CoV-2 infection to prevent IgG-level competition observed with hypergammaglobulinemia in other infections (Okoko et al., 2001; Farquhar et al., 2005). In contrast to HA-specific antibody transfer in COVID^–^ mothers, SARS-CoV-2-specific antibody transfer trended higher with increased IgG levels (Figure 5F), likely due to the accompanying increase of SARS-CoV-2 antibodies along with overall increased IgG levels.

To understand the influence of IgG levels on SARS-CoV-2-antibody transfer, the pairwise influence of elevated IgG and all individual Fc-features on transplacental transfer was examined. While previous reports have shown that galactosylation is important for transfer across the placenta, IgG levels appeared to selectively augment G1S1F, NK cell-activating (CD107a and MIP-1b), afucosylated/agalactosylated (G0), fucosylated/agalactosylated (G0F), and G2S1F transfer, among the top 10 IgG-co-correlates in predicting transfer (Figure 5G). Additionally, increasing IgG levels tracked with improved transfer of complement- (ADCD) enhancing antibodies, and FCGR3 transfer was found among the top transfer predictors, highlighting the importance of this FcR in placental sieving.

To specifically explore the impact of IgG-levels on shifting Fc-glycan transfer during SARS-CoV-2 infection, the influence of IgG level combined with galactosylation was compared to the glycan effect on transfer alone. IgG level alone was a poor predictor of transfer (Figure 5H, bottom). In contrast to patterns seen in pertussis-specific antibody transfer (Jennewein et al., 2019), SARS-CoV-2-specific agalactosylated/afucosylated Fc glycans were enriched in cord blood. Interestingly, in the presence of elevated IgG, all glycans transferred more efficiently, albeit agalactosylated/afucosylated glycans were the most associated with IgG1 spike transfer (Figure 5H), pointing to IgG level enhancement of all S-specific antibodies with a slight preference for afucosylated antibodies. These data suggest that elevated IgG levels may compensate for poor SARS-CoV-2-specific antibody transfer.

Previous studies suggest that the placenta preferentially transfers digalactosylated antibodies (Kibe et al., 1996; Jennewein et al., 2019; Borghi et al., 2020). However, SARS-CoV-2 antibody transfer consistently pointed to enhanced transfer of agalactosylated/afucosylated spike-specific antibodies (Figures 4E, 5G, and 5H). To understand this selection based on fucose content, we next dissected the fucosylation pattern on antibodies with varying degrees of galactosylation (Figure 5I). Whereas the level of fucosylation was conserved across galactosylated bulk antibodies in COVID^+^ and COVID^–^ mothers (Figures 3B and 3C), agalactosylated spike-specific antibodies showed a trend toward a decreased proportion of fucosylation (Figure 5I, left). Conversely, fucosylation was significantly higher on galactosylated antibody subpopulations (Figure 5I, middle and right). Thus, although maternal spike-specific antibodies exhibit enhanced galactosylation (Figures 3D and 3E), these glycans also contain high levels of fucose, which may reduce the likelihood of transfer in the setting of collaborative FcRn and FCGR3A transplacental transfer (Pincetic et al., 2014).

### Increased FCGR3A and FcRn colocalization in SARS-CoV-2 infection

**Figure 6:**
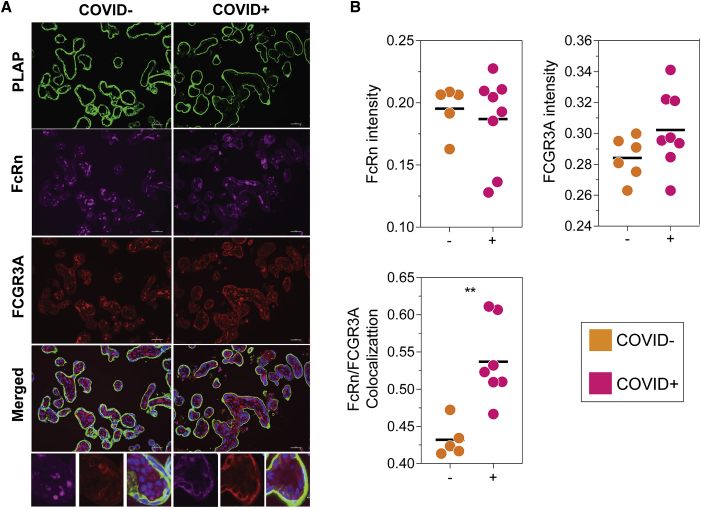
Increased FcRn/FCGR3A colocalization following SARS-CoV-2 infection (A) Placental tissue sections from COVID^+^ and COVID^–^ mothers were stained for FcRn (purple). FCGR3A (red), and placental alkaline phosphatase (PLAP, green), a trophoblast marker. (B) The dot plots represent the intensity of FcRn, FCGR3, and FcRn/FCGR3 colocalization in placental tissues. Significance was determined by an unpaired Student’s t test with Welch’s correction, ^∗∗^p < 0.01.

Preferential transfer of agalactosylated/afucosylated SARS-CoV-2 antibodies points to a critical role for FCGR3A selection during placental transfer. To probe whether placental Fc receptor expression patterns were altered by maternal SARS-CoV-2 infection, we quantified the levels of FcRn, FCGR3A, and the colocalization of these receptors, which have been previously identified as instrumental in placental antibody transfer (Jennewein et al., 2019). While no difference was observed in FcRn expression, a slight increase in FCGR3A was observed in placentas from COVID^+^ mothers (Figures 6A and 6B). Moreover, colocalization of the two receptors was significantly increased in placentas from COVID^+^ mothers (Figures 6A and 6B), suggesting that enhanced FCGR3A expression and FCGR3A/FcRn colocalization may compensate for galactose changes and promote the specific selection of afucosylated antibodies able to drive enhanced NK cell function. Taken together, these data suggest that elevated IgG levels may limit the transfer of total antibodies but facilitate the transfer of current-infection-specific antibodies recently generated by the pregnant mother. Enhanced FCGR3A/FcRn colocalization may be a compensatory third-trimester pregnancy-specific immune mechanism, allowing the placenta is able to continue to select functionally optimized antibodies that drive enhanced NK cell activation to deliver the best immunity possible to the neonate.

## Discussion

Specific populations are at greatest risk in the SARS-CoV-2 pandemic, including individuals with significant medical comorbidities, the elderly, racial and ethnic minorities, patients of low socioeconomic status, and pregnant women (Ellington et al., 2020; Mehra et al., 2020; Richardson et al., 2020; Wadhera et al., 2020; Goldfarb et al., 2020). While SARS-CoV-2 infection is not known to be widespread in newborns and infants, this pediatric population is particularly vulnerable to severe disease upon SARS-CoV-2 infection (Dong et al., 2020), with substantial morbidity and death reported (Liguoro et al., 2020; Mithal et al., 2020; Ovalı, 2020). These reports, coupled with the potential for neonates and infants to develop the severe Kawasaki-like disease called multisystem inflammatory syndrome in children (MIS-C) (Feldstein et al., 2020), and high viral loads in this age group that may act as a reservoir of viral spread (Bialek et al., 2020; Yonker et al., 2020), highlight the urgency of understanding factors that contribute to disease outcomes in this population. While initial pregnancy literature focused on detecting vertical transmission and placental infection (Alzamora et al., 2020; Hosier et al., 2020; Patanè et al., 2020; Penfield et al., 2020; Vivanti et al., 2020), it has become increasingly clear that vertical transmission and placental infection are rare events (Flaherman et al., 2020; Edlow et al., 2020). As our data and others demonstrate, this does not eliminate risk for SARS-CoV-2 infection for neonates and infants, who may be more vulnerable to infection due to relative lack of maternally transferred humoral immunity, especially relative to other vaccinatable respiratory infections. Despite the disproportionate susceptibility of pregnant women and their newborns to infections, they are among the last to receive vaccines due to required enhanced safety stringency. Understanding the gaps in the immune response in these populations may provide key insights for the rational selection and design of therapeutics and vaccines able to selectively protect this vulnerable population.

Here, we report that, while bulk and non-SARS-CoV-2-specific antibody transfer to the cord is intact in COVID^+^ mothers, SARS-CoV-2-specific antibody transfer is significantly compromised in the third trimester of pregnancy, related to perturbed Fc glycosylation. This deficiency is not observed when infection occurs in the second trimester of pregnancy, pointing to a vulnerability arising from third-trimester infection and providing critical insights for pregnancy-specific disease pathogenesis and vaccine design. Changes in antibody glycosylation have been noted following infections, with unique glycan profiles emerging on pathogen-specific antibodies (Ackerman et al., 2013). These changes in Fc glycosylation give disease-specific antibodies the capacity to recruit innate immune effector functions, aimed at controlling the pathogen more effectively (Ackerman et al., 2013; Mahan et al., 2016). As expected, overall bulk, HA- and PTN-specific Fc-binding profiles were unaltered across the COVID^+^ and COVID^–^ mothers, given that these humoral immune responses are not leveraged during SARS-CoV-2 infection. Thus, SARS-CoV-2 infection during pregnancy does not disrupt the general antibody glycome, despite pregnancy itself being associated with changes in antibody glycosylation (van de Geijn et al., 2009; Bondt et al., 2013). Conversely, SARS-CoV-2-specific antibodies did exhibit perturbed glycosylation, potentially aimed at leveraging immune functions to protect the mother from the virus. Alterations in placental expression of Fc-receptors that facilitate the transfer of maternal antibodies to the neonate were also noted. Overall, these data point to a delicate, previously unappreciated balance during third-trimester pregnancy, whereby infection results in both changes in pathogen-specific antibodies positioned to protect the dyad, and changes within the placenta, aimed at compensating for Fc changes that otherwise would render transfer inefficient.

While compromised transfer has been noted in prior studies of *de novo* infection during pregnancy (Perret et al., 2005; Castanha et al., 2019), the specific antibody Fc-alterations and compensatory placental changes that occur, aimed at maintaining antibody transfer to infants, have not been described. The perturbations in SARS-CoV-2 antibody glycan profiles resulted in both reduced transfer and altered glycan-transfer sieving. The neonates received higher levels of agalactosylated, sialylated, and bisected SARS-CoV-2-specific antibodies, in contrast to the highly galactosylated nature of bulk antibody transfer. Among SARS-CoV-2-specific antibodies, agalactosylated/fucosylated (G0F) antibodies were retained in the mothers, but agalactosylated/afucosylated (G0) antibodies were selectively transferred to the cord. Given that fucose reduces binding to FCGR3A (Subedi and Barb, 2016), which regulates antibody transfer in the placenta (Jennewein et al., 2019), this discrepancy in the transfer of agalactosylated antibodies highlights a selective-transfer based on fucose content. Spike-specific antibodies with Fc galactosylation had increased fucosylation compared to bulk antibodies with Fc-galactosylation, potentially explaining the decreased transfer efficiency of SARS-CoV-2-specific antibodies. Consistent with the role of fucose in regulating NK cell activity and FCGR3A binding (Alter et al., 2018), the selective transfer of SARS-CoV-2-specific NK cell-activating antibodies pointed to conserved principles of enhanced functional antibody transfer. Additionally, SARS-CoV-2 infection was associated with a selective increase in FCGR3A and FCGR3A/FcRn colocalization, potentially driving enhanced selection and transfer of these afucosylated antibodies. These findings may be relevant for developing COVID^–^ vaccine strategies, with vaccine regimens able to drive high levels of afucosylated and galactosylated antibodies in order to optimize placental transfer.

While perturbed placental transfer of infection-associated antibodies were observed across two independent third-trimester infection cohorts, antibody transfer normalized following second-trimester infection, suggesting that inflammation-induced alterations in SARS-CoV-2-specific-glycan profiles may resolve over time from infection. These data suggest that changes in Fc glycosylation proximal to the time of infection may be the most significant contributor to poor placental transfer. Whether antibodies with perturbed glycosylation profiles will also be observed following third-trimester vaccination with a *de novo* immunogen remains unclear. These data point to a critical nuance in antibody-transfer dynamics, where pre-existing antibodies may be transferred exponentially during the third trimester, but *de novo* produced antibodies may transfer more effectively if induced earlier in pregnancy. Thus, understanding how *de novo* antibody transfer varies by trimester may point to critical windows in pregnancy that may be most desirable for induction of antibodies through vaccination to optimize protection for both the mother and her infant.

Reduced maternal-to-neonatal placental antibody transfer has been noted in the setting of maternal chronic and acute infection, including HIV, dengue, and malaria infection, among others (Okoko et al., 2001; Farquhar et al., 2005; Atwell et al., 2016). While SARS-CoV-2 infection is largely transient and not typically associated with hypergammaglobulinemia, elevated IgG titers were noted in COVID^+^ women. HA- and PTN-antibody transfer was largely conserved across COVID^+^ and COVID^–^ dyads, suggesting that the ability of the placenta to transfer antibodies was overall not compromised. However, IgG titer was associated with restricted HA-specific antibody transfer in COVID^–^ dyads and enhanced SARS-CoV-2 antibody transfer in COVID^+^ dyads, the latter likely by simply augmenting overall antibody levels including SARS-CoV-2-specific antibodies. Together with increased placental colocalization of FCGR3A and FcRn on syncytiotrophoblasts, the presence of higher levels of SARS-CoV-2 antibodies may increase the probability of delivering the most functional SARS-CoV-2 antibodies to the neonate, namely, afucosylated antibodies that activate the anti-viral NK cell response.

Despite the unfavorable transfer profile of SARS-CoV-2 antibodies during *de novo* third-trimester infection, and the known increased risk of hospitalization and ICU admission when disease occurs in neonates and infants, severe infection in infants and neonates still remains relatively rare compared to adults (Zeng et al., 2020; Ovalı, 2020; Yang et al., 2020; Dong et al., 2020; Kim et al., 2020). Low prevalence of neonatal infection despite suboptimal antibody protection is likely attributable to a combination of low expression of ACE2 receptors in nasal and other respiratory epithelia (Bunyavanich et al., 2020), behavioral modifications such as maternal masking and hand hygiene, and some degree of maternal-to-cord anti-SARS-CoV-2 antibody transfer in neonates. However, as infections rise, increasing numbers of newborns will be delivered to women infected across the trimesters of pregnancy, and new vulnerabilities as well as new principles of antibody transfer may emerge in this population that will be among the last to receive vaccination. Defining the rules of placental antibody selection and transfer in the setting of infection, marked by specific antibody Fc-alterations and linked Fc-receptor expression profile changes in the placenta, may provide critical insights for next generation vaccine development to ensure that both pregnant women and their neonates are empowered with robust levels of immunity against SARS-CoV-2 and beyond.

### Limitations of study

There are several limitations in this study. First, since these samples were collected during the first months of the SARS-CoV-2 pandemic, the sample size of COVID^+^ mothers was small. However, we were able to validate inefficient antibody transfer in the third trimester in a second independent cohort. We were also able to isolate the vulnerability to the third trimester of pregnancy by the comparison to a cohort of women infected in the second trimester. Whether the first trimester is also a time of vulnerability can only be defined in future studies. While these data are contrary to pre-existing vaccine transfer efficiency data that suggest that third-trimester vaccination is associated with the highest level of placental transfer (Ben-Hur et al., 2005; Abu Raya et al., 2014), these observations may be exclusively related to boosting of pre-existing immunity for repeat vaccination. Differences between the transfer of antibodies generated by *de novo* infection or vaccination and repeat vaccination can be addressed in animal studies.

Second, given that some of the COVID^+^ mothers were asymptomatic, we were unable to precisely define days since symptom onset for these individuals. Thus, the time from infection analysis was conservatively estimated based on time of testing. More accurate symptom-related timing analysis and repeated sampling during pregnancy may provide enhanced resolution on how the evolving immune response contributes to antibody transfer to neonates.

Finally, antibodies may be transferred to neonates via breast milk that arm mucosal tissues against viral infection. Future studies aimed at profiling the humoral immune response across gestation and across transfer mechanisms (placental, mammary) may provide a full picture of the pregnancy-related vulnerabilities that must be addressed via vaccination. Overall, the data presented here point to a deficiency in antibody transfer from third-trimester infection that may extend beyond SARS-CoV-2 infection. Understanding how SARS-CoV-2-specific antibody transfer is impacted by gestational age at infection and Fc glycosylation profiles has profound implications for vaccine design and vaccination strategies in order to provide the greatest protection for both mother and neonate.

## STAR★Methods

### Key resources table

**Table:** 

REAGENT or RESOURCE	SOURCE	IDENTIFIER
**Antibodies**		

anti-CD66b-Pacific blue	BioLegend	CAT # 305112
APC-Cy7 Mouse Anti-Human CD16.	BD Biosciences	CAT # 557758; RRID:AB_396853
PE-Cy7 Mouse Anti-Human CD56	BD Biosciences	CAT # 557747; RRID: AB_2033963
PE MIP-1b Mouse anti-Human	BD Biosciences	CAT # 550078; RRID:AB_393549
Pacific Blue Mouse Anti-Human CD3	BD Biosciences	CAT # 558117; RRID:AB_1595437
Human IgG-heavy and light chain Antibody	Bethyl Laboratories	CAT # A80-119P
FITC Goat IgG anti-C3	MP Biomedicals	CAT # 855385
Mouse Anti-Human IgG1-Fc PE	Southern Biotech	CAT # 9054-09
Mouse Anti-Human IgG2-Fc PE	Southern Biotech	CAT # 9060-09
Mouse Anti-Human IgG3-Hinge PE	Southern Biotech	CAT # 9210-09
Mouse Anti-Human IgG4-Fc PE	Southern Biotech	CAT # 9200-09
Mouse Anti-Human IgA1-Fc PE	Southern Biotech	CAT # 9130-09
Mouse Anti-Human IgM-Fc PE	Southern Biotech	CAT # 9020-09
Anti-Placental alkaline phosphatase (PLAP) antibody [ALPP/870]	Abcam	CAT # ab212383
Anti-FCGRT/FCRN antibody - Extracellular domain	Abcam	CAT # ab193148
Anti-CD16 antibody	Leica Biosystems	CAT # NCL-L-CD16
Goat anti-Mouse IgG2a	Invitrogen	CAT # A-21133
Goat anti-mouse IgG2b	Invitrogen	CAT # A-21141
Goat anti-rabbit	Abcam	CAT # ab150080

**Bacterial and virus strains**		

SARS-CoV-2-S pseudovirus with a luciferase reporter	This paper	N/A

**Chemicals, peptides, and recombinant proteins**		

SARS-CoV-2 S	Obtained from the lab of Dr. Eric Fischer	N/A
SARS-CoV-2 RBD	Obtained from the lab of Dr. Aaron Schmidt	N/A
SARS-CoV-2 N	Aalto Bio Reagents	CAT # CK 6404-b
SARS-CoV-2 S1	Sino Biological	Cat # 40591-V08H
SARS-CoV-2 S2	Sino Biological	CAT # 40590-V08B.
Pertactin from B. Pertussis (69 kDa Protein)	List Biological Laboratories	CAT # 187
A/Michigan/45/2015 (H1N1)	Immunetech	CAT # IT-003-00105ΔTMp
B/Phuket/3073/2013	Immunetech	CAT # IT-003-B11ΔTMp
A/Singapore/INFIMH-16-0019/2016	Immunetech	CAT # IT-003-00434ΔTMp
Human Fc receptors	Produced at the Duke Human. Vaccine Institute, . {Boesch, 2014 #15}	N/A
Streptavidin-R-Phycoerythrin	Prozyme	CAT # PJ31S
FIX&Perm Cell Permeabilization Kit	Life Tech	CAT # GAS001S100; CAT # GAS002S100
Human IL-15 Recombinant Protein, eBioscience	ThermoFisher Scientific	CAT # BMA31
1-Step Ultra TMB-ELISA substrate solution	ThermoFisher Scientific	CAT # 34029
IDEZ-Protease	New England Biolabs	CAT # P0770S
Brefeldin A.	Sigma Aldrich	CAT # B7651
GolgiStop	BD Biosciences	CAT # 554724
Luciferase Assay Reagent	Promega	CAT # E1483
Background Sniper	Biocare medical	CAT # BS966L
Vector True View	Vector Laboratories	CAT # SP8400-15
DAPI	ThermoFisher Scientific	CAT # D13060
Vectashield mounting medium	Vector Laboratories	CAT # H-1000
streptavidin magnetic beads	NEB	CAT # S1420S

**Critical Commercial Assays**		

BirA-500: BirA biotin-protein ligase standard. reaction kit	Avidity	CAT # BirA500
RosetteSep Human NK Cell Enrichment Cocktail	Stem Cell Technologies	CAT # 15065
Steady-Glo Luciferase Assay	Promega	CAT # E2510
GlycanAssure HyPerformance APTS Kit.	ThermoFisher Scientific	CAT # A33952

**Experimental models: cell lines**		

THP-1 Cells	ATCC	CAT # TIB-202; RRID: CVCL_0006

**Software and algorithms**		

GraphPad Prism	GraphPad	https://www.graphpad.com/scientific-software/prism/
Intellicyt ForeCyt Software	Sartorious	https://intellicyt.com/products/software/
CellProfiler software	Broad Institute	https://cellprofiler.org/
R programming language	Version 3.6.1	https://www.r-project.org/
GlycanAssure software	ThermoFisher Scientific	https://www.thermofisher.com/us/en/home/life-science/bioproduction/contaminant-and-impurity-testing/glycanAssure-Glycan-analysis.html.

**Other**		

FluoSpheres NeutrAvidin-Labeled Microspheres, 1.0 μm, yellow-green fluorescent (505/515), 1% solids	Invitrogen	CAT # F8776
FluoSpheres NeutrAvidin-Labeled Microspheres, 1.0 μm, red fluorescent (505/515), 1% solids	Invitrogen	CAT # F8775
MagPlex microspheres	Luminex corporation	CAT # MC12001-01, MCI12040-01, MCI10077-01

### Resource availability

#### Lead contact

Further information and requests for resources and reagents should be directed to and will be fulfilled by the Lead Contact, Galit Alter (galter@partners.org).

#### Materials availability

This study did not generate new unique reagents.

#### Data and code availability statement

The dataset generated during this study is available upon reasonable request.

### Experimental model and subject details

#### Sample Cohort

Maternal and cord blood plasma samples from 22 mother-cord dyads who were previously infected with SARS-CoV-2 and 34 uninfected, contemporaneously-enrolled mother-neonate dyads were collected at Massachusetts General Hospital (MGH) and Brigham and Women’s Hospital (BWH) at the time of delivery (Shook et al.*,* 2020). Placental samples collected from a subset of enrolled participants were fixed at the time of delivery and utilized for immunohistochemical analyses. Maternal and cord blood plasma from an additional third trimester validation cohort comprised of 28 mother-neonate dyads and a second trimester cohort comprised of 29 mother-neonate dyads was collected from participants enrolled at the same institutions. Individuals were tested for SARS-CoV-2 by real-time reverse-transcriptase–polymerase-chain-reaction (RT-PCR) of nasopharyngeal swab. Neonates born to COVID+ mothers were tested for SARS-CoV-2 via RT-PCR of nasopharyngeal swab, as per hospital policy. Maternal disease severity was determined based on published criteria from the Society for Maternal-Fetal Medicine and the National Institutes of Health. All enrolled participants provided informed consent. This study was approved by the MGH-BWH (Mass General Brigham) Institutional Review Board.

#### Cell Lines

THP-1 cells (ATCC), an acute monocytic leukemia cell, were grown at 37°C, 5% CO2 in RPMI with 10% fetal bovine serum, L-glutamine, penicillin/streptomycin, HEPES and beta-mercaptoethanol.

#### Primary Immune Cells

Human neutrophils and NK cells were isolated from fresh peripheral blood. Peripheral blood was collected by the MGH Blood Bank or by the Ragon Institute from healthy volunteers. All volunteers were over 18 years of age and gave signed consent. Samples were deidentified before use. The study was approved by the MGH Institutional Review Board. Human neutrophils were maintained in R10 (RPMI with 10% fetal bovine serum, L-glutamine, penicillin/streptomycin and HEPES) and grown at 37°C, 5% CO2 for the duration of the assay. Human NK cells were rested overnight in R10 supplemented with IL-15 at 37°C, 5% CO2 and maintained in R10 for the duration of the assay.

### Method details

#### Functional Assays

Antibody-dependent cellular phagocytosis (ADCP), antibody-dependent neutrophil phagocytosis (ADNP) and antibody-dependent complement deposition (ADCD) were performed as previously described (Ackerman et al., 2011; Fischinger et al., 2019; Karsten et al., 2019). Antigens used for functional assays were: SARS-CoV-2 RBD (kindly provided by Aaron Schmidt), SARS-CoV-2 S (kindly provided by Eric Fischer), SARS-CoV-2 N (Aalto Bio Reagents) and a mix of HA A/Michigan/45/2015 (H1N1), HA A/Singapore/INFIMH-16-0019/2016 (H3N2), HA B/Phuket/3073/2013 (Immunetech).

Antigen was biotinylated using Sulfo-NHS-LC-LC biotin (Thermo) and desalted using Zeba Columns (Thermo). Yellow-green (for ADCP and ADNP) or red (for ADCD) were incubated with biotinylated antigen for 2 hours at 37°C or overnight at 4°C. Coupled beads were washed twice with 0.01% BSA in PBS and resuspended at a concentration of 10ug/mL. To form immune complexes, antigen-coupled beads were added to 96-well plates with an equal volume of appropriately diluted plasma (1:10 for ADCD, 1:100 for ADCP and ADNP) and incubated for 2 hours at 37°C. Immune complexes were washed following incubation. For ADCP, 1.25x10^5^ THP-1 cells/mL were added to immune complexes and incubated for 16-18 hours at 37°C. Following the incubation, cells were fixed with 4% PFA. For ADNP, white blood cells were isolated from whole blood from healthy donors using ammonium-chloride potassium lysis to lyse red blood cells. 2.5x10^5^ cells/mL were added to immune complexes and incubated for 1 hour at 37°C. Neutrophils were stained with an anti-CD66b PacBlue (Biolegend) detection antibody and fixed with 4% PFA. For ADCD, lyophilized guinea pig complement (Cedarlane) was resuspended according of manufacturer’s recommendations and diluted in gelatin veronal buffer supplemented with calcium and magnesium (Boston BioProducts). The diluted complement was added to immune complexes and incubated at 37°C for 20 minutes. C3 deposition was detected using an anti-C3 fluorescein-conjugated goat IgG fraction detection antibody (Mpbio).

The NK cell activation assay was performed as described previously (Boudreau et al., 2020). Briefly, ELISA plates were coated with 2 ug/mL of antigen, incubated for 2 hours at 37°C, washed with PBS, and blocked with 5% BSA overnight at 4°C. NK cells were isolated from fresh peripheral blood (MGH Blood Bank) using RosetteSep (Stem Cell Technologies) and Ficoll separation. NK cells were rested overnight in media supplemented with IL-15. To form immune complexes, diluted plasma (1:50) was added to coated ELISA plates and incubated for 2 hours at 37°C. Plates were washed with PBS and NK cells were at a concentration of 2.5x10^5^ cells/mL in media supplemented with GolgiStop (BD), Brefeldin A (BFA, Sigma Aldrich) and anti-CD107a PE-Cy5 (BD). Plates were incubated for 5 hours at 37°C. After incubation, NK cells were fixed with Perma A (Life Tech), and stained for the surface markers anti-CD3 PacBlue (BD), anti-CD16 APC-Cy5 (BD), and anti-CD56 PE-Cy7 (BD). NK cells were then permeabilized with Perm B (Life Tech) and stained with anti-MIP-1β PE (BD) antibody.

Fluorescence for the functional assays was acquired using an iQue (Intellicyt). For phagocytosis assays, a phagocytic score was calculated using the following formula: (percentage of bead-positive cells) x (GeoMean of MFI of bead-positive cells)/10,000. ADCD is reported as median MFI of C3 deposition. NK cells were gates as CD3-, CD16+, CD56+ cells and NK cell activity was determined as the percent of NK cells positive for CD107a and MIP-1b.

#### Luminex

A multiplexed Luminex assay was used to determine relative titer of antigen-specific isotypes, subclasses, and FcR binding, as previously described (Brown et al., 2017). For this assay, the following antigens were used: SARS-CoV-2 RBD (kindly provided by Aaron Schmidt), SARS-CoV-2 S (kindly provided by Eric Fischer), SARS-CoV-2 N (Aalto Bio Reagents). SARS-CoV-2 S1 (Sino Biological), SARS-CoV-2 S2 (Sino Biological), pertussis pertactin (List Reagents) and a mix of HA A/Michigan/45/2015 (H1N1), HA A/Singapore/INFIMH-16-0019/2016 (H3N2), B/Phuket/3073/2013 (Immunetech). Antigens were covalently linked to carboxyl-modified Magplex© Luminex beads using Sulfo-NHS (N-hydroxysulfosuccinimide, Pierce) and ethyl dimethylaminopropyl carbodiimide hydrochloride (EDC). Antigen-coupled microspheres were blocked, washed, resuspended in PBS, and stored at 4C.

To form immune complexes, appropriately diluted plasma (1:100 for IgG2/3, 1:500 for IgG1 and FcRn, 1:1000 for all other FcRs) was added to the antigen-coupled microspheres, and plates were incubated overnight at 4°C, shaking at 700 rpm. The following day, plates were washed with 0.1% BSA 0.02% Tween-20. PE-coupled mouse anti-human detection antibodies (Southern Biotech) were used to detect antigen-specific antibody binding. For the detection of FcR binding, Avi-Tagged FcRs (Duke Human Vaccine Institute) were biotinylated using BirA500 kit (Avidity) per manufacturer’s instructions. Biotinylated FcRs were tagged with PE and added to immune complexes. Fluorescence was acquired using an Intellicyt iQue, and relative antigen-specific antibody titer and FcR binding is reported as Median Fluorescence Intensity (MFI).

#### Glycan analysis

Spike antigen was biotinylated using Sulfo-NHS LC-LC-biotin (Thermo) and coupled to streptavidin magnetic beads (NEB). Samples were heat inactivated by incubation at 56°C for 60 minutes. Samples were spun at 20,000 g for 10 minutes and the supernatant was taken for use in the assay. Heat-inactivated plasma and cord blood was incubated for 30 minutes with uncoupled streptavidin magnetic beads to remove non-specific bead binding. Sample was then removed from the magnetic beads and added to protein-coupled magnetic beads for 1 hour at 37°C to form immune complexes. The immune complexes were washed and incubated with IDEZ (NEB) to cleave off the Fc from spike-specific antibodies overnight at room temperature. The Fc were deglycosylated using PNGase and labeled with APTS per manufacturer’s protocol (Glycan Assure APTS kit). Glycans were analyzed using a 3500xL genetic analyzer (Applied Biosystems). N-glycan fucosyl, afucosyl and afucosyl fucosyl mixed libraries were also run with the samples to enable identification of glycan species. The relative frequency of glycan specifies was determined using GlycanAssure software.

#### ELISA

To determine the IgG titer against SARS-CoV-2 RBD, S, N, or influenza HA, ELISA plates were coated with antigen at 500ng/mL and incubated for 30 minutes at room temperature. To determine total IgG levels, ELISA plates were coated with maternal plasma or cord blood diluted 1:100 and incubated for 30 minutes at room temperature. After coating, plates were washed and blocked with a 1% blocking solution for 30 minutes at room temperature. For the antigen specific ELISA, maternal plasma or cord blood were diluted 1:100 and added to the plates and plates were incubated for 30 minutes at room temperature. Plates were washed and IgG was detected using anti-human IgG-horseradish peroxidase (HRP) (Bethyl Laboratories). The ELISA was developed using 1-Step Ultra TMB (Invitrogen) and stopped with sulfuric acid. Absorbance was read at 450 nm and subtracted using a 570 nm reference.

#### Immunofluorescence

For co-labeling immunofluoresence experiments, placenta tissue sections were rehydrated in an alcohol series after deparaffinization in xylene. Antigen retrieval was performed by boiling in 10 mM sodium citrate (pH 6.0) for 30 minutes and cooled at room temperature before blocking for 15 minutes with Background Sniper (Biocare Medical). Samples were then incubated in primary antibodies diluted in 5% bovine serum albumin (BSA) for 1.5 hours at room temperature (Placental Alkaline Phosphatase (PLAP: Abcam, ab212383) - 1:1000, Neonatal Fc Receptor (FcRn: Abcam, ab193148) – 1:100, CD16 (CD16: Leica Biosystems NCL-L-CD16) – 1:100. The slides were washed in PBS Tween 0.1% and incubated in fluorescently-conjugated secondary antibodies (1:500 in 5% BSA – Goat anti-Mouse IgG2a (A-21133), Goat anti-mouse IgG2b (A-21141), Goat anti-rabbit (Abcam, ab150080)). Finally, the slides were treated with Vector True View to eliminate red blood cells and background (Vector Laboratories, SP8400-15), treated with DAPI (ThermoFisher, D1360) and coverslipped with Vectashield mounting medium (Vector Laboratories, H-1000).

CellProfiler software was used to quantify CD16/FcRn colocalization as well as their respective intensities. Briefly, RGB pictures were converted to gray and placental villi were selected to avoid measuring background. Following background removal, the pictures were filtered using the function CorrectIlluminationCalculate and aligned to evaluate colocalization. Finally, CD16 (FCGR3A) and FcRn intensities in the placental villi previously delimitated were used to quantify colocalization. Unpaired Student’s t test was used to compare control and SARS-CoV-2 positive placental expression using Prism software (Graphpad version 8.0).

### Quantification and statistical analysis

#### Univariate Analyses

All univariate plot were visualized and analyzed using Graphpad software, version 8.0. Univariate plots show the average of two replicates of the functional assays and Luminex assay. Transfer ratios were calculated by dividing infant antibody by mother antibody levels. Transfer ratios may appear unusually high when maternal antibodies are very low.

#### Multivariate Analyses

Multivariate analyses included glycan, Luminex, and functional antibody measurements. Only Luminex-derived features with a median raw score greater than 10,000 were included in the analyses to prevent noise from obscuring biological signal. All features were centered and scaled to unit variance. Multilevel partial least square discrimination analysis (mPLSDA) was performed for all classification models between paired mother and chord samples. mPLSDA generates models that focus on within-pair variance (Westerhuis et al., 2010). For regression models, orthogonal partial least square regression (OPLSR) was performed on mother samples without multilevel preprocessing. An orthogonal PLSDA was applied to the data after feature reduction via elastic net regularization and variable selection. The elastic net algorithm balances the minimization of features included in the model and optimization of model performance (Zou and Hastie, 2005). ElasticNet parameters were first optimized using a tuneLength = 10 and leave-one-out cross validation. The final reduced feature sets compromised of the features included in greater than 80% of the 100 rounds of variable selection. An orthogonal PLSDA was implemented using the R ‘ropls’ Bioconductor package (orthI = 1; PredI = 1). All analyses in R were performed using R (version 4.0.0).

#### Correlation Networks

Pairwise spearman’s correlation tests were performed comparing each feature in the full dataset to the reduced feature set included in the final OPLS-DA model. Correlations with coefficients > 0.75 and p value < 0.05 were inputted into Cytoscape (version 3.8.0) to produce correlation networks. Feature type (ie- Luminex, glycan, functional) was denoted by distinct node color.
